# Amorpha-4,11-diene synthase: a key enzyme in artemisinin biosynthesis and engineering

**DOI:** 10.1007/s42994-021-00058-x

**Published:** 2021-07-30

**Authors:** Jin Quan Huang, Xin Fang

**Affiliations:** 1 National Key Laboratory of Plant Molecular Genetics, Institute of Plant Physiology and Ecology/CAS Center for Excellence in Molecular Plant Sciences, Chinese Academy of Sciences, Shanghai, 200032 People’s Republic of China; 2 State Key Laboratory of Phytochemistry and Plant Resources in West China, Kunming Institute of Botany, Chinese Academy of Sciences, Kunming, 650204 Yunnan People’s Republic of China

**Keywords:** Amorpha-4,11-diene Synthase, Artemisinin, Catalytic mechanism, Metabolic engineering, Synthetic biology

## Abstract

Amorpha-4,11-diene synthase (ADS) catalyzes the first committed step in the artemisinin biosynthetic pathway, which is the first catalytic reaction enzymatically and genetically characterized in artemisinin biosynthesis. The advent of ADS in *Artemisia annua* is considered crucial for the emergence of the specialized artemisinin biosynthetic pathway in the species. Microbial production of amorpha-4,11-diene is a breakthrough in metabolic engineering and synthetic biology. Recently, numerous new techniques have been used in ADS engineering; for example, assessing the substrate promiscuity of ADS to chemoenzymatically produce artemisinin. In this review, we discuss the discovery and catalytic mechanism of ADS, its application in metabolic engineering and synthetic biology, as well as the role of sesquiterpene synthases in the evolutionary origin of artemisinin.

## Introduction

**Fig. 1:**
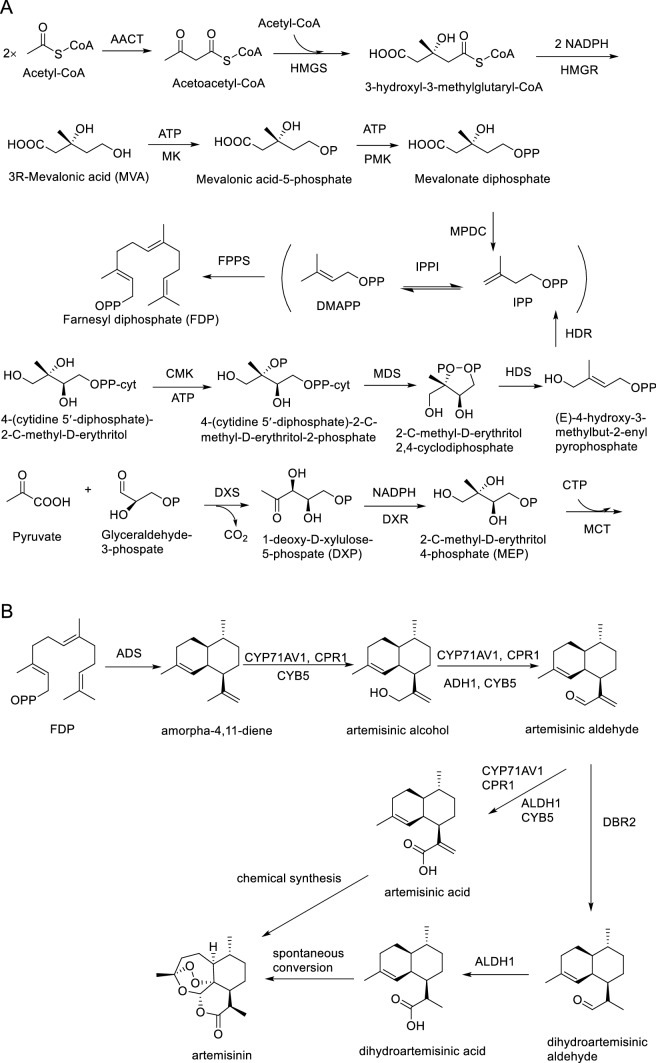
Proposed artemisinin biosynthetic pathway in *A. annua*. A. Carbon flow from MVA (in the cytosol) and MEP (in the chloroplast) pathways to form FDP. AACT, acetyl-coenzyme transferase; HMGS and HMGR, 3-hydroxy-3-methylglutaryl-CoA synthase and reductase; MK, 3R-mevalonic acid kinase; PMK, mevalonic acid-5-phosphate kinase; MPDC, mevalonate 5-pyrophosphate decarboxylase; DXS and DXR, 1-deoxy-D-xylulose 5-phosphate synthase and reductase; MCT, 2-C-methyl-D-erythritol 2,4-cyclodiphosphate synthase; CMK, 4-diphosphocytidyl-2-C-methyl-D-erythritol kinase; MDS, 2-C-methyl-D-erythritol 2,4-cyclodiphosphate synthase; HDS and HDR, 4-hydroxy-3-methylbut-2-enyl pyrophosphate synthase and reductase; IPPI, isopentenyl pyrophosphate isomerase; FPPS, farnesyl pyrophosphate synthase. B. Production of artemisinin *in planta* and by biological methods. ADS, armorpha-4,11-diene synthase; CYP71AV1, cytochrome P450 monooxygenase; CPR1, cytochrome P450 reductase 1; CYB5, cytochrome b5 monooxygenase; ALDH1, aldehyde dehydrogenase; DBR2, artemisinic aldehyde delta-11(13)-double bond reductase. Enzymes marked in red improved the efficiency of different oxidation steps in yeast (Paddon et al. 2013)

Artemisinin is a well-known antimalarial drug against chloroquine-resistant strains of *Plasmodium falciparum* (Chen and Xu 2016; Wang et al. 2019). In 2002, the World Health Organization recommended artemisinin-based combinatorial therapies as the first-line treatment for uncomplicated malaria. *Artemisia annua* L. is the only natural source of artemisinin, which is biosynthesized and accumulated in the glandular trichome cells of the plant. A recent study has proved that artemisinin is also produced in the non-glandular trichome cells (Judd et al. 2019). The low content of artemisinin in *A. annua* (0.1–1.0% of dry weight) makes its plant-derived production insufficient for global requirements (Ikram and Simonsen 2017). Recent advances in metabolic engineering and synthetic biology have enabled higher yield of artemisinin in microbial or plant heterologous hosts by engineering the artemisinin pathway genes in these hosts. However, complete understanding of artemisinin biosynthesis is still required (Fig. 1).

The first step toward the elucidation of the artemisinin biosynthetic pathway began in 1999, when amorpha-4,11-diene synthase (ADS) was purified from *A. annua* and functionally characterized (Bouwmeester et al. 1999). Since then, studies have been extensively conducted to understand the artemisinin biosynthetic pathway and its evolutionary origin and to develop metabolic engineering and biosynthetic methods for its production. Notably, stable and high-yielding production of artemisinic acid was established in *Saccharomyces cerevisiae* and chemical conversion of artemisinic acid effectively yielded artemisinin (Paddon and Keasling 2014). Recently, the production of amorpha-4,11-diene has become a touchstone technique because of its important role in metabolic engineering and synthetic biology (Choi et al. 2016; Orsi et al. 2020; Redding-Johanson et al. 2011; Shukal et al. 2019; Wang et al. 2013; Yuan and Ching 2014, 2015a, 2015b, 2016). Furthermore, the substrate promiscuity of ADS was used to develop a chemoenzymatic strategy for artemisinin production (Demiray et al. 2017). The importance of ADS still attracts considerable research attention.

Because advances in the investigation of artemisinin biosynthesis as well as in metabolic engineering or synthetic biology for artemisinin production have been previously reviewed (Farhi et al. 2013; Ikram and Simonsen 2017; Kung et al. 2018; Xie et al. 2016), herein, we mainly focus on the discovery, catalytic mechanism, and engineering of ADS, as well as the impact of the emergence of *ADS* in the evolutionary origin of artemisinin biosynthetic pathway in this review.

## Characterization, catalytic mechanism, and evolutionary origin of ADS

Structurally, artemisinin is an endoperoxide sesquiterpene lactone in which the basic carbon skeleton is constructed from farnesyl diphosphate (FDP) by a sesquiterpene synthase. In 1999, a native ADS protein was purified from *A. annua* and functionally characterized, suggesting that ADS may catalyze the first rate-determining step in artemisinin biosynthesis (Bouwmeester et al. 1999). Several groups have isolated terpene synthase genes from *A. annua* and bacterially expressed them in *Escherichia coli*, resulting in the identification of ADS (Chang et al. 2000; Mercke et al. 2000; Wallaart et al. 2001) and other terpene synthases, such as (3*R*)-linalool synthase (Jia et al. 1999), 8-epicedrol synthase (Hua and Matsuda 1999; Mercke et al. 1999), and β-caryophyllene synthase (Cai et al. 2002), from *A. annua*. Overexpression and downregulation of *ADS* in *A. annua* plants resulted in increased and reduced artemisinin content *in planta*, respectively, providing direct genetic evidence of the involvement of ADS in artemisinin biosynthesis (Alam and Abdin 2011; Catania et al. 2018; Han et al. 2016; Ma et al. 2009, 2015). These studies have paved the way for further investigation of artemisinin biosynthesis.

ADS is a class I terpenoid synthase (TPS) belonging to the plant TPS-a subgroup (Salmon et al. 2015). It contains conserved DDXXD (DDTYD) and NSE/DTE (NDLMTHKAE) ion-binding motifs (Chang et al. 2000; Mercke et al. 2000). Accordingly, the effects of divalent metal ions, such as Mg^2+^, Mn^2+^, Co^2+^, Ni^2+^, Zn^2+^, and Cu^2+^, on enzyme activity were tested. No activity was reported when Ni^2+^, Zn^2+^, and Cu^2+^ were used, and the enzyme activity was lower with Mn^2+^ and Co^2+^ than with Mg^2+^ (Picaud et al. 2005). ADS showed 36% and 41% amino acid sequence identity with tobacco 5-*epi*-aristolochene synthase (TEAS) and cotton ( +)-δ-cadinene synthase, respectively (Chang et al. 2000). Similar to the crystal structure of TEAS, ADS also has an N-terminal glycosyl hydrolase domain and a C-terminal catalytic domain (Mercke et al. 2000).

The catalytic mechanism of ADS has attracted continued interest. Generally, the catalytic mechanism of a class I TPS involves the initial ionization of the substrate diphosphate group, electrophilic cyclization, deprotonation, or capture of a nucleophile, and finally, the release of neutral products (Christianson 2017). Methods used to investigate mechanistic details involve labeled substrates and mutant enzymes and include X-ray crystallography and quantum chemical study (Faraldos et al. 2012). A catalytic model of ADS was suggested by the observation of TEAS, in which FDP binding placed Phe^525^ next to Trp^271^ to form an extended aromatic box, and the carbocation intermediates were stabilized by the nucleophilicity of the Trp^271^ indole ring. The ionization of FDP was facilitated by the positive charges of Arg^262^ and Arg^440^ with the help of divalent metal cations coordinated by the DDXXD motif. Other motifs including the Arg^10^–Pro^11^ pair and the Asp^444^–Tyr^520^–Asp^524^ triad were also conserved in ADS, but their function was not experimentally investigated (Chang et al. 2000; Mercke et al. 2000).

**Fig. 2:**
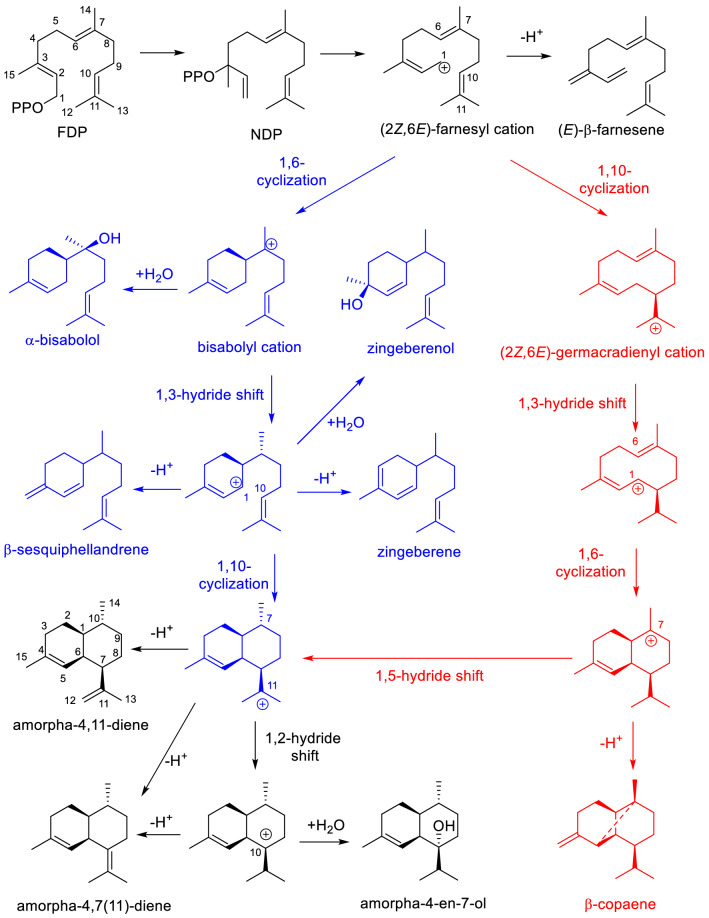
Proposed cyclization mechanisms for the formation of amorpha-4,11-diene by ADS. Reactions starting from the initial 1,6-ring closure of FDP and the generation of bisabolene-type by-products are highlighted in blue. Steps proceeding through the initial 1,10-ring closure and corresponding by-products are marked in red

The bicyclic structure of amorpha-4,11-diene is formed by an initial 1,6 or 1,10 cyclization of FDP involving a bisabolyl or (2Z,6E)-germacradienyl cation, respectively (Chang et al. 2000). The recombinant ADS expressed in *E. coli* produces the by-products *β*-sesquiphellandrene, *α*-bisabolol, zingiberene, and zingibernol, supporting the involvement of a bisabolyl cation in the cyclization mechanism (Mercke et al. 2000; Picaud et al. 2005). By using deuterium-labeled FDP (labeled at C-1) as a chemical probe, two study groups independently found that H-1 migrated to the original C-7 of FDP (C-10 of amorpha-4,11-diene). They deduced the occurrence of the initial 1,6 cyclization because the initial 1,10-ring closure led to the shift of H-1 to C-11 (Kim et al. 2006; Picaud et al. 2006). However, a subsequent 1,5-hydride shift could also allow the migrating H-1 to relocate to the original C-7 of FDP (Fig. 2), indicating that these two cyclization mechanisms cannot be determined by using labeled substrates. Indeed, a quantum chemical study concluded that the 1,5-hydride shift is feasible, supporting the occurrence of the initial 1,10 cyclization for the catalytic mechanism of ADS (Hong and Tantillo 2010). Recently, the ADS Q518L variant was reported to generate the initial 1,10 cyclization product *β*-copaene in addition to amorpha-4,11-diene (Fig. 2), implying that ADS uses both initial 1,6 and 1,10 cyclization mechanisms to produce amorpha-4,11-diene (Huang et al. 2021).

**Fig. 3:**
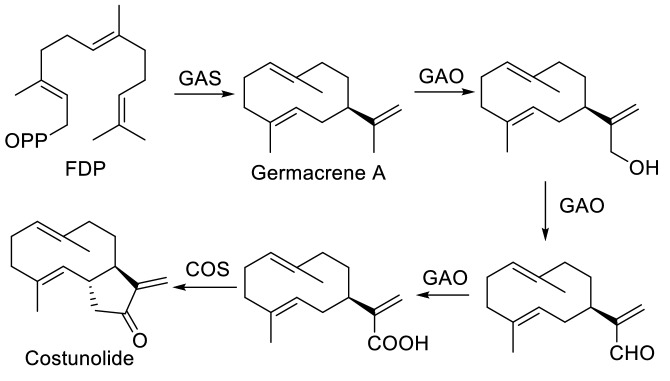
Asteraceae sesquiterpene lactone biosynthetic pathway. GAS, germacrene A synthase; COS, costunolide synthase

New terpenoid biosynthetic pathways usually initiate from the emergence of functional TPS/CYP gene pairs (Boutanaev et al. 2015), but the sequence of their occurrence is not fixed. Regarding the evolutionary emergence of the artemisinin biosynthetic pathway in *A. annua*, Nguyen et al. (2010) suggested that the occurrence of *ADS* is a dominant event mainly shaped by an Y374L mutation in its progenitor (Salmon et al. 2015). They found that in all major subfamilies of Asteraceae, germacrene A oxidase (GAO) is conserved when producing germacrene A acid, the key intermediate of the Asteraceae sesquiterpene lactone biosynthetic pathway (Fig. 3). Remarkably, GAO uses amorpha-4,11-diene as the substrate to produce artemisinic acid, whereas CYP71AV1 or amorpha-4,11-diene oxidase (AMO) is inactive to germacrene A. Thus, they hypothesized that the advent of ADS activity in *A. annua* removed GAO from the Asteraceae sesquiterpene lactone biosynthetic pathway and eventually replaced it with AMO. In addition, sesquiterpene lactones derived from germacrene A are absent in *A. annua* but are present in other *Artemisia* species (Bertea et al. 2006). The promiscuity of GAO and the specificity of AMO were further confirmed by the ability of GAO to oxidize several sesquiterpenes, including germacrene D, 5-epi-aristolochene, valencene, δ-cadinene, α- and δ-guaienes, and valerenadiene to corresponding sesquiterpene acids, whereas AMO showed negligible activities (Nguyen et al. 2019). Similarly, orthologs of *CYP71AV1* (94% amino acid identity) from the *Artemisia* genus (e.g., *A. afra* and *A. absinthium*) converted amorpha-4,11-diene to artemisinic alcohol (Komori et al. 2013). However, ADS homologs from other *Artemisia* species (e.g., *A. absinthium, A. kurramensis,* and *A. maritima*) did not produce amorpha-4,11-diene (Muangphrom et al. 2016); a homologous synthase from *A. maritima* produced amorphen-4,11-ol (Muangphrom et al. 2018). Although further investigation is required, accumulated data is in favor of the hypothesis that artemisinin production in *A. annua* is attributed to the emergence of *ADS*.

## Metabolic engineering of ADS

Microbial production of artemisinin is a milestone in the development of synthetic biology (Kung et al. 2018), which initiated from the expression of *ADS* in engineered *E. coli* (Martin et al. 2003). To increase amorpha-4,11-diene production, several improvements have been made (Table 1), including the development of a two-phase partitioning bioreactor (Newman et al. 2006); identifying and enhancing the production of rate-limiting enzymes (Fig. 1), such as *MK*, *PMK*, *HMGS*, *HMGR*, and *ADS* (Anthony et al. 2009; Ma et al. 2011; Redding-Johanson et al. 2011; Tsuruta et al. 2009); increasing the flux of 1-deoxy-D-xylulose-5-phosphate by engineering the phosphoenolpyruvate-dependent phosphotransferase system (*PTS*; Zhang et al. 2013, 2015); systematically optimizing transcription and translation in *E. coli* (Shukal et al. 2019); and constructing multienzyme complexes in *E. coli* (Wei et al. 2020). Among these improvements, assembling and modulating efflux pumps in *E. coli* are vital because the accumulation of antimicrobial amorpha-4,11-diene in *E. coli* inhibited cell growth (Zhang et al. 2016; Wang et al. 2013). Collectively, the highest production of amorpha-4,11-diene was 27.4 g/L (Tsuruta et al. 2009).

**Table 1:** Biosynthetic and metabolic engineering approaches to produce amorpha-4,11-diene

Construction	Organism	Amorphadiene	References
Engineering codon-optimized ADS and mevalonate pathway from *S. cerevisiae* in *E. coli*	*E. coli*	24 mg/L	Martin et al. (2003)
Enhancing production of rate-limiting enzymes MK and ADS	*E. coli*	300 mg/L	Anthony et al. (2009)
Introducing more active HMG-CoA synthase and HMG-CoA reductase	*E. coli*	27.4 g/L	Tsuruta et al. (2009)
Enhancing production of rate-limiting enzymes MK and PMK	*E. coli*	500 mg/L	Redding-Johanson et al. (2011)
Introducing more active HMG-CoA reductase	*E. coli*	700 mg/L	Ma et al. (2011)
Engineering efflux pumps	*E. coli*	363 mg/L	Wang et al. (2013)
Deleting PTS	*E. coli*	182 mg/L	Zhang et al. (2013)
Engineering PTS and GGS	*E. coli*	201 mg/L	Zhang et al. (2015)
Efflux transporter engineering	*E. coli*	150 mg/L	Zhang et al. (2016)
Systematically optimizing transcription and translation in *E. coli*	*E. coli*	30 g/L	Shukal et al. (2019)
Plasmid integration of *ADS* into yeast	*S. cerevisiae*	0.6 mg/L	Lindahl et al. (2006)
Inserting *ADS* into yeast genome	*S. cerevisiae*	0.1 mg/L	Lindahl et al. (2006)
Overexpressing *tHMGR*, *ERG20*, and *upc2-1*, and downregulating *ERG9*	*S. cerevisiae*	153 mg/L	Ro et al. (2006)
Increasing copy number of *ADS* in yeast	*S. cerevisiae*	781 mg/L	Ro et al. (2008)
Engineering codon-optimized *ADS* in *S. cerevisiae*	*S. cerevisiae*	123 mg/L	Kong et al. (2009)
Integrating *HMG1*, *FDPS*, and *ADS* into yeast mitochondria	*S. cerevisiae*	20 mg/L	Farhi et al. (2011a)
Overexpressing every enzyme of MVA pathway	*S. cerevisiae*	41 g/L	Westfall et al. (2012)
Downregulating *ERG9* and fusing *ADS* with *FPPS*	*S. cerevisiae*	25 mg/L	Baadhe et al. (2013)
Combinatorial genome integration of MVA pathway genes in yeast	*S. cerevisiae*	64 mg/L	Yuan and Ching (2014)
Knockout genes outside isoprenoid pathway but improving isoprenoid fluxes	*S. cerevisiae*	54.5 mg/L	Sun et al. (2014)
Dynamic control of the expression of *ERG9*	*S. cerevisiae*	350 mg/L	Yuan and Ching (2015a)
Assembling MVA pathway genes into yeast chromosomes and reducing *ERG9* expression	*S. cerevisiae*	500 mg/L	Yuan and Ching (2015b)
Integrating MVA pathway genes and *ADS* into yeast mitochondria	*S. cerevisiae*	427 mg/L	Yuan and Ching (2016)
Expressing ADS in *N. tabacum*	*N. tabacum*	1.7 ng/g FW	Wallaart et al. (2001)
Targeting FPS and ADS in plastids	*N. tabacum*	25 μg/g FW	Wu et al. (2006)
Introducing *tHMGR*, *FPS*, and *ADS* into *N. benthamiana*	*N. benthamiana*	6.2 μg/g FW	Van Herpen et al. (2010)
Targeting FPS and ADS in plastids	*N. tabacum*	4 μg/g FW	Zhang et al. (2011)
Introducing *tHMGR* from yeast and *ADS, CPR, CYP71AV1*, and *DBR2* into *N. tabacum*	*N. tabacum*	827 ng/g FW	Farhi et al. (2011b)
Introducing whole artemisinin pathway genes into *N. tabacum* chloroplasts	*N. tabacum*		Fuentes et al. (2016)
Engineering MVA and artemisinin pathway genes in *N. tabacum* chloroplasts, nuclei, and mitochondria	*N. tabacum*	60 μg/g DW	Malhotra et al. (2016)
Engineering *ADS* in *P. patens*	*Physcomitrella patens*	200 mg/L	Ikram et al. (2017)
Engineering *dxs, idi*, and *ADS* in *B. subtilis*	*B. subtilis*	20 mg/L	Zhou et al. (2013)
Chromosomally integrated *GFP*-*ADS*, *FPPS*, and a plasmid-encoded synthetic operon carrying MEP pathway genes	*B. subtilis*	416 mg/L	Pramastya et al. (2020)
Engineering MEP pathway genes and *ADS* in cyanobacteria	*Synechococcus elongatus PCC 7942*	19.8 mg/L	Choi et al. (2016)
Engineering codon-optimized *ADS* in *S. avermitilis*	*S. avermitilis*	30 μg/L	Komatsu et al. (2010)
Engineering *ADS* in *R. sphaeroides*	*R. sphaeroides*	56.4 mg/L	Orsi et al. (2020)

Although the production of amorpha-4,11-diene in engineered *E. coli* was successful, several enzymes are necessary to complete the transformation of amorpha-4,11-diene to artemisinin, in which the cytochrome P450 CYP71AV1 catalyzes the first oxidation reaction (Teoh et al. 2006). N-terminal modified CYP71AV1 was heterologously expressed in *E. coli* (Chang et al. 2007); however, the functional expression of plant P450 in *E. coli* is extremely challenging because intracellular membrane structures are absent.

Yeast is considered a reliable host for the expression of plant P450. To facilitate the expression of *CYP71AV1*, two groups independently engineered yeast to produce amorpha-4,11-diene in 2006. Lindahl et al. (2006) expressed *ADS* in yeast using plasmids and chromosomal integration resulting in 0.6 mg/L and 0.1 mg/L amorpha-4,11-diene production, respectively, whereas Ro et al. (2006) obtained 153 mg/L of amorpha-4,11-diene by introducing *ADS* into yeast that was simultaneously engineered by the overexpression of truncated *HMGR*, FPPS (*ERG20*), and an activated allele of the *UPC2* transcription factor (*upc2-1*) as well as the downregulation of the expression of squalene synthase (*ERG9*). Since then, other metabolic engineering methods have been used in yeast to increase amorpha-4,11-diene production (Table 1). These methods include mutating the *ADS* gene to the yeast-conform variant (Kong et al. 2009), using a high-copy plasmid system to express *ADS* in yeast (Ro et al. 2008), downregulating the expression of *ERG9* and fusing *ADS* with *FPPS* (Baadhe et al. 2013; Yuan and Ching 2015a), integrating the combinatorial genome of mevalonate (MVA) pathway genes in yeast (Yuan and Ching 2014), using knockout genes outside the isoprenoid pathway but improving isoprenoid fluxes (Sun et al. 2014), assembling MVA pathway genes into yeast chromosomes and reducing *ERG9* expression (Yuan and Ching 2015b), and expressing MVA pathway genes and *ADS* into yeast mitochondria (Farhi et al. 2011b; Yuan and Ching 2016). By overexpressing every enzyme of the MVA pathway, the production of amorpha-4,11-diene reached 41 g/L (Westfall et al. 2012).

By expressing artemisinin pathway genes in microbial hosts, current biosynthetic methods only produced artemisinic acid. However, introducing artemisinin pathway genes in *Nicotiana spp*. resulted in the heterologous production of artemisinin, suggesting a metabolic engineering application of these plants in the production of artemisinin (Kram and Simonsen 2017). Initially, *ADS* was expressed in *N. tabacum* to characterize its function, but it only yielded 1.7 ng/g (fresh weight) of amorpha-4,11-diene (Wallaart et al. 2001). Methods similar to those for the microbial production of amorpha-4,11-diene (Table 1), such as coexpressing MVA pathway genes and targeting *ADS* into mitochondria, chloroplasts, or plastids, were used to improve the accumulation of amorpha-4,11-diene (Farhi et al. 2011a; Fuentes et al. 2016; Malhotra et al. 2016; van Herpen et al. 2010; Wu et al. 2006; Zhang et al. 2011). Using the moss *Physcomitrella patens* as a heterologous host avoided the glycosylation of pathway intermediates (Ikram et al. 2017, 2019), and this effect was similar to the expression of artemisinin pathway genes in the chloroplasts, nuclei, and mitochondria of *N. tabacum* (Fuentes et al. 2016; Malhotra et al. 2016).

The success of microbial production of amorpha-4,11-diene in *E. coli* and yeast has promoted engineering other organisms for amorpha-4,11-diene production as proof-of-concept studies. For example, *Bacillus subtilis* was chosen because of its rapid growth rate and safe status (Pramastya et al. 2020; Song et al. 2021; Zhou et al. 2013), and cyanobacteria were engineered as biosolar cell factories for the photosynthetic conversion of CO_2_ to amorpha-4,11-diene (Choi et al. 2016). The industrial microorganism *Streptomyces avermitilis* was genetically engineered to produce amorpha-4,11-diene but none of its major endogenous secondary metabolites (Komatsu et al. 2010). *Rhodobacter sphaeroides* was used to test the growth-independent production of isoprenoids such as amorpha-4,11-diene (Orsi et al. 2020). Another strategy to produce high-value natural products is in vitro metabolic engineering, which has been applied for the production of amorpha-4,11-diene and some inhibitors of ADS such as ATP and pyrophosphate were identified (Chen et al. 2013; 2016).

## Protein engineering and chemoenzymatic application of ADS

The above approaches in improving amorpha-4,11-diene production often involve the enhancement of the efficiency and production of rate-limiting enzymes in metabolic flux. However, protein engineering of ADS itself for higher catalytic efficiency has not been attempted. Engineering ADS is important because it catalyzes the first committed step in the formation of the artemisinin carbon skeleton but has poor catalytic activity. The classic metabolic engineering approach of increasing enzyme concentration to increase the production of target molecules is often limited by inherent low enzyme activity, particularly for TPS, which has 30 times lower enzyme activity than the central metabolism enzymes, which is also the case for ADS (Bar-Even et al. 2011). Protein engineering to improve the catalytic efficiency of TPS is a promising solution to this problem, which includes rational and non-rational engineering (Leonard et al. 2010). Non-rational engineering is based on error-prone PCR that introduces random mutations to a target gene, followed by the screening of clones for the desired function. Because of the lack of a high-throughput assay for screening mutant libraries, this method for engineering TPS is difficult (Lauchli et al. 2013). Thus, rational engineering of TPS is an alternative approach, which requires both knowledge of catalytic processes and understanding of the three-dimensional structure of TPS.

In 2013, *A. annua* α-bisabolol synthase (BOS) was isolated and functionally characterized to understand its crystal structure (Li et al. 2013). It shares 82% amino acid sequence identity and the bisabolyl cation as a common intermediate with ADS, providing a basis to find the active residues involved in ADS catalysis. After partially elucidating ADS catalysis, a T399S ADS variant that showed twofold higher turnover rate (*k*_cat_) because of accelerated product release was found (Li et al. 2013). This inspired continuous investigation for rational engineering of ADS. As mentioned earlier, the ADS catalytic mechanism involves sequential 1,6 and 1,10 cyclization (Fig. 2). By using BOS (single 1,6 cyclization) and germacrene A synthase (single 1,10 cyclization) from *A. annua* as reference (Bertea et al. 2006), *A. annua* phylogeny-based site-directed substitutions were performed. This led to the identification of seven residues in ADS controlling the whole cyclization process of amorpha-4,11-diene formation and a double mutation T399S/T447S that tripled *k*_cat_ (Fang et al. 2017). Interestingly, the ADS T296V variant abolished the cyclization to the bisabolyl cation (Fang et al. 2017; Li et al. 2016; Abdallah et al. 2018). Four residues L374, L404, L405, and L439 were collectively responsible for the 1,10 cyclization, and T399 and T447 catalyzed the regioselective deprotonation and product release of ADS (Fang et al. 2017). To further identify active site residues, homology models of ADS based on a BOS variant (Abdallah et al. 2016) and TEAS (Eslami et al. 2017) were constructed. The root-mean-square deviation values between the BOS and TEAS models were 2.35 Å and 0.302 Å, respectively. Guided by the BOS model, extensive mutations were performed, leading to the identification of several residues influencing ADS catalysis. These residues included R262 for binding the PPi group; W271, Y519, and F525 for stabilizing intermediate carbocations; G400, G439, and L515 for the 1,10-ring closure; T399 for regioselective deprotonation; and W271 as an active site catalytic base. A double mutation T399S/H448A that improved *k*_cat_ by 5 times was also reported (Abdallah et al. 2016, 2018). Similarly, by using the TEAS model, residues identified were involved in FDP binding and determining the fate of the allylic carbocation intermediate. These residues included Y519, D444, W271, N443, T399, R262, V292, G400, and L405, which largely overlapped with those reported by other groups (Eslami et al. 2017). Collectively, these studies have provided insight into the sequence–function relationships of ADS and have impacted the industrial production of artemisinin by microbial fermentation.

In addition to catalytic efficiency, product specificity of ADS is another target of protein engineering. Heterologous expression of ADS in *E. coli* yielded 89% of amorpha-4,11-diene (Newman et al. 2006), whereas an in vitro enzymatic reaction led to 80% of amorpha-4,11-diene in addition to 15 by-products (Picaud et al. 2005), and one of these by-products, amorpha-4-en-11-ol, was recently found to exist as an epimer of 6(*R*/*S*)-amorpha-4-en-11-ol (Huang et al. 2021). In contrast, the recombinant ADS expressed in *N. benthamiana* produced 97% of amorpha-4,11-diene and 3% of amorpha-4,7(11)-diene in vitro (Kanagarajan et al. 2012), suggesting that CYP71AV1 may not be exposed to the above 14 by-products produced by ADS expressed in *E. coli* (except for amorpha-4,7(11)-diene) *in planta*. Recently, it was demonstrated that CYP71AV1 could not use any of the 15 by-products as substrates, including amorpha-4,7(11)-diene and amorpha-4-en-7-ol, which are structurally similar to amorpha-4,11-diene, suggesting an overlooked issue to improve the fidelity of heterologously expressed ADS for more effective production of this artemisinin precursor by fermentation in *E. coli* (Huang et al. 2021).

**Fig. 4:**
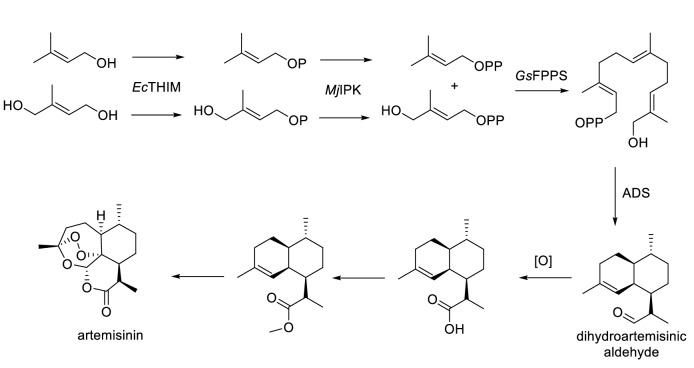
Chemoenzymatic approach to synthesize artemisinin. *Ec*THIM, *E. coli* hydroxyethylthiazole kinase; *Mj*IPK, *Methanocaldococcus jannaschii* isopentenyl phosphate kinase; *Gs*FPPS, *Geobacillus stearothermophilus* farnesyl pyrophosphate synthase

By exploiting the substrate promiscuity of ADS, a chemoenzymatic strategy was recently developed for artemisinin production. Demiray et al. (2017) found that ADS accepted chemically synthesized 12-hydroxy-FDP as the substrate and converted it to dihydroartemisinic aldehyde. When the enzymatic reaction was performed using high-performance counter current chromatography, the yield of dihydroartemisinic aldehyde increased from 20 to 60% and the reaction time reduced about tenfold (Huynh et al. 2020). In a few chemical steps, a high yield of artemisinin was obtained from this intermediate (Tang et al. 2018). Furthermore, by using the substrate promiscuity of kinases and FPPS, 12-hydroxy-FDP was enzymatically synthesized in quantitative yield (Johnson et al. 2020). On reversing the oxidation order, the entire route was complementary to the biosynthetic approach (Fig. 4).

## Conclusion and perspectives

ADS catalyzes the first committed step in the artemisinin biosynthetic pathway. Therefore, any approach using synthetic biology and metabolic engineering to synthesize artemisinin heterologously should start with the expression of ADS. Commercial scale production of semi-synthetic artemisinin was developed based on the progress of synthetic biology for artemisinin production. Evolutionarily, the emergence of ADS in *A. annua* essentially shapes a specialized artemisinin pathway from the costunolide pathway. Collectively, insights from these approaches have improved our knowledge and understanding of secondary metabolism biosynthesis, metabolic engineering, and synthetic biology.

However, some questions still remain unanswered. For example, bacterial systems used to express ADS produce 10% of by-products that cannot be used by downstream CYP71AV1. This reduces the efficiency of metabolic flux for artemisinin production by microbial fermentation and requires further investigation. Regarding the catalytic mechanism of ADS, the current mechanism was proposed based on mutation and labeled substrate experiments but was not supported by quantum chemical studies. Thus, X-ray crystallography data is needed. Besides, the three-dimensional structure of ADS expressed in *E. coli* and *in planta* will unravel the molecular basis for product promiscuity of recombinant ADS from *E. coli*.

More importantly, a question that needs to be answered is whether the conversion from artemisinic acid to artemisinin is dependent or independent of enzymes *in planta*. Although chemical transformation of these compounds is feasible in plant hosts—artemisinic acid is readily converted to artemisinin, such conversion is not feasible in microbial hosts. This observation suggests a missing enzymatic link between dihydroartemisinic acid and artemisinin in *A. annua*.
