# Correction to: Amorpha-4,11-diene synthase: a key enzyme in artemisinin biosynthesis and engineering

**DOI:** 10.1007/s42994-021-00059-w

**Published:** 2021-09-21

**Authors:** Jin Quan Huang, Xin Fang

**Affiliations:** 1 National Key Laboratory of Plant Molecular Genetics, Institute of Plant Physiology and Ecology/CAS Center for Excellence in Molecular Plant Sciences, Chinese Academy of Sciences, Shanghai, 200032 People’s Republic of China; 2 State Key Laboratory of Phytochemistry and Plant Resources in West China, Kunming Institute of Botany, Chinese Academy of Sciences, Kunming, 650204 Yunnan People’s Republic of China

## Correction to: aBIOTECH 10.1007/s42994-021-00058-x

The original version of this article unfortunately contained a mistake. The spelling of Jin Quan Huang’s name was incorrect. The corrected author list is given above. The original article has been corrected.

